# Pharmacokinetics, bioavailability, and plasma protein binding study of glytrexate, a novel multitarget antifolate

**DOI:** 10.3389/fphar.2022.1001308

**Published:** 2022-10-04

**Authors:** Jiahong Xiang, Mengqi Wu, Jianchao Wang, Mengmeng Lin, Mengmeng Sun, Xin Li, Ruijuan Xing, Ran Guo, Jianmin Gu, Tao Lyu, Lei Wang, Xiaowei Shi

**Affiliations:** ^1^ Key Laboratory of Innovative Drug Research and Evaluation in Hebei Province, School of Pharmaceutical Sciences, Hebei Medical University, Shijiazhuang, China; ^2^ Department of Medicinal Chemistry, School of Pharmaceutical Sciences, Hebei Medical University, Shijiazhuang, China; ^3^ Department of General Practice, The Second Hospital, Hebei Medical University, Shijiazhuang, China

**Keywords:** pharmacokinetics, bioavailability, glytrexate, plasma protein binding, antifolate

## Abstract

Glytrexate, developed by our team, as a novel multitarget folate antagonist, has inhibitory effects on a variety of cancer cell types, especially KB tumor cells (IC_50_ 0.078 nM), and thus has antitumor drug development prospects. However, its pharmacokinetics and plasma protein binding properties remain unknown. In this study a selective and sensitive liquid chromatography-tandem mass spectrometry (LC‒MS/MS) method was developed and verified to facilitate biological analysis. The bioanalysis method was applied to evaluate the stability, plasma protein binding, and pharmacokinetics of glytrexate. Glytrexate is more stable in human plasma than in rat plasma and in human liver microsomes. The binding of glytrexate to human plasma proteins was higher than that to rat plasma proteins, both of which were less than 30%, suggesting that glytrexate may be at a higher concentration at the pharmacologic target receptor(s) in tissues. Pharmacokinetic characteristics were determined by noncompartmental analysis after administration of single oral (12.5, 25 and 50 mg/kg) and intravenous (2 mg/kg) doses in rats. According to the rat oral pharmacokinetic characteristics, glytrexate had linear dynamics in a dose range of 12.5–50 mg/kg and a poor oral bioavailability of 0.57–1.15%. The investigation revealed that the intravenous half-life, AUC, and C_max_ of glytrexate were higher than those of pemetrexed. Pemetrexed is generally produced as an injection preparation. This provides ideas for the development of glytrexate formulations. Therefore, glytrexate injection has clinical application prospects compared to oral administration. This study provides a basis for further investigations into the pharmacological effects and clinical uses of glytrexate.

## Graphical Abstract

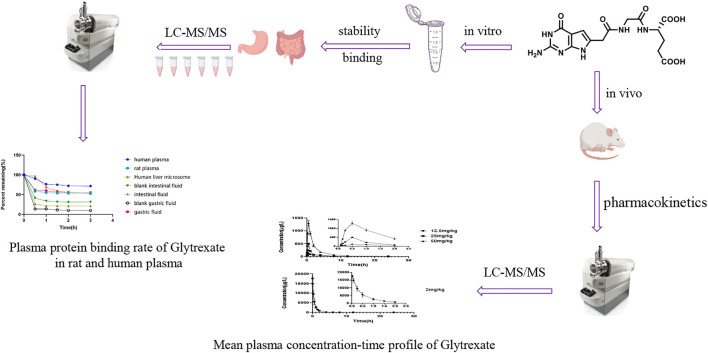

## 1 Introduction

Antifolates that target folate metabolism have been crucial in the treatment of cancer, infectious disorders, and chronic inflammatory diseases. Antifolates have a lengthy history and are widely used. Aminopterin, the first antifolate applied in the clinic, was first reported in the New England Journal of Medicine in June 1948 (Farber and Diamond, 1948). This medication was replaced with methotrexate in the early 1950s, which is attributed to the unpredictability of aminopterin-related toxicity (Nichol, 1954). The second antifolate, pemetrexed, was authorized for use in 2004 for the treatment of mesothelioma and later non-small cell lung cancer, more than 50 years after the launch of methotrexate (Rollins and Lindley, 2005). The search for new antifolates with enhanced characteristics and better activities is still desirable.

By preventing folate-dependent one-carbon biosynthetic and methylation processes, antifolates prevent cellular proliferation (Paz-Ares et al., 2003; Sigmond et al., 2003; Visentin et al., 2012). Thymidylate synthase (TS), dihydrofolate reductase (DHFR), glycinamide ribonucleotide formyltransferase (GARFTase), and 5-aminoimidazole-4-carboxamide ribonucleotide formyltransferase (AICARFTase) are involved in purine and pyrimidine synthesis, and interfering with their function results in the inhibition of DNA and RNA synthesis (Thorndike et al., 1990; Takimoto, 1996; Caperelli and Giroux, 1997; Jarmula, 2010; Lele et al., 2016; Fales et al., 2017). Medications with a single target may interfere with that target but not modify the entire disease, whereas drugs with numerous targets can control several elements of the disease and modulate synergistic targets to increase efficacy and minimize side effects. Thus, a multitargeted antifolate may help ameliorate the problem of drug resistance (Takemura et al., 1997; Purcell and Ettinger, 2003).

In our previous study, a novel series of 6-substituted pyrrolo [2,3-d] pyrimidines were developed and synthesized as possible anticancer drugs targeting both thymidylate and purine nucleotide biosynthesis (Xing et al., 2017). Glytrexate, (S)-2-{2-[2-(2-amino-4-oxo-4,7-dihydro-3H-pyrrolo [2,3-d]pyrimidin-6-yl)-acetylamino]-acetylamino}-pentanedioic acid (1), was one of a series of compounds identified as a multitarget inhibitor of TS, GARFTase, and AICARFTase, and exhibited antiproliferative effects in a series of tumor cell lines including KB, SW620, and MCF7 cells (Xing et al., 2017). Its inhibitory action on KB tumor cells was at a nanomolar level (IC_50_ 0.078 nM), and its efficacy was 140-fold higher than the positive control drug pemetrexed (IC_50_ 0.07 μM) (Xing et al., 2017). Therefore, glytrexate is a potential preclinical development candidate. However, the druggability of glytrexate, including its pharmacokinetics, plasma binding properties, and stability in plasma and liver microsomes, has not been fully elucidated.

The development of bioanalytical methods and the characterization of pharmacokinetic properties are requirements for preclinical pharmacological studies of candidate drugs. Pharmacokinetic assessments are a prerequisite for elucidating drug mechanisms, which is essential for lead identification and optimization in drug discovery and preclinical development. Pharmacokinetic modeling contributes to a comprehensive and accurate estimation of dose‒response-time data, and thus provides valuable references for understanding and improving drug efficacy, optimizing clinical dosage, reducing toxicity and adverse effects, and identifying clinical implications. In terms of pharmacokinetics and pharmacodynamics, the plasma protein binding rate is a crucial measure that has gained widespread acceptance in drug development. However, there is not enough information on the *in vivo* and *in vitro* profiles of glytrexate. Here, we developed an LC‒MS/MS method to conduct assays of glytrexate in biological samples. The validated method was applied to investigate the pharmacokinetics, absolute bioavailability, plasma protein binding rate, and stability of glytrexate.

## 2 Experimental

### 2.1 Materials and animals

**FIGURE 1:**
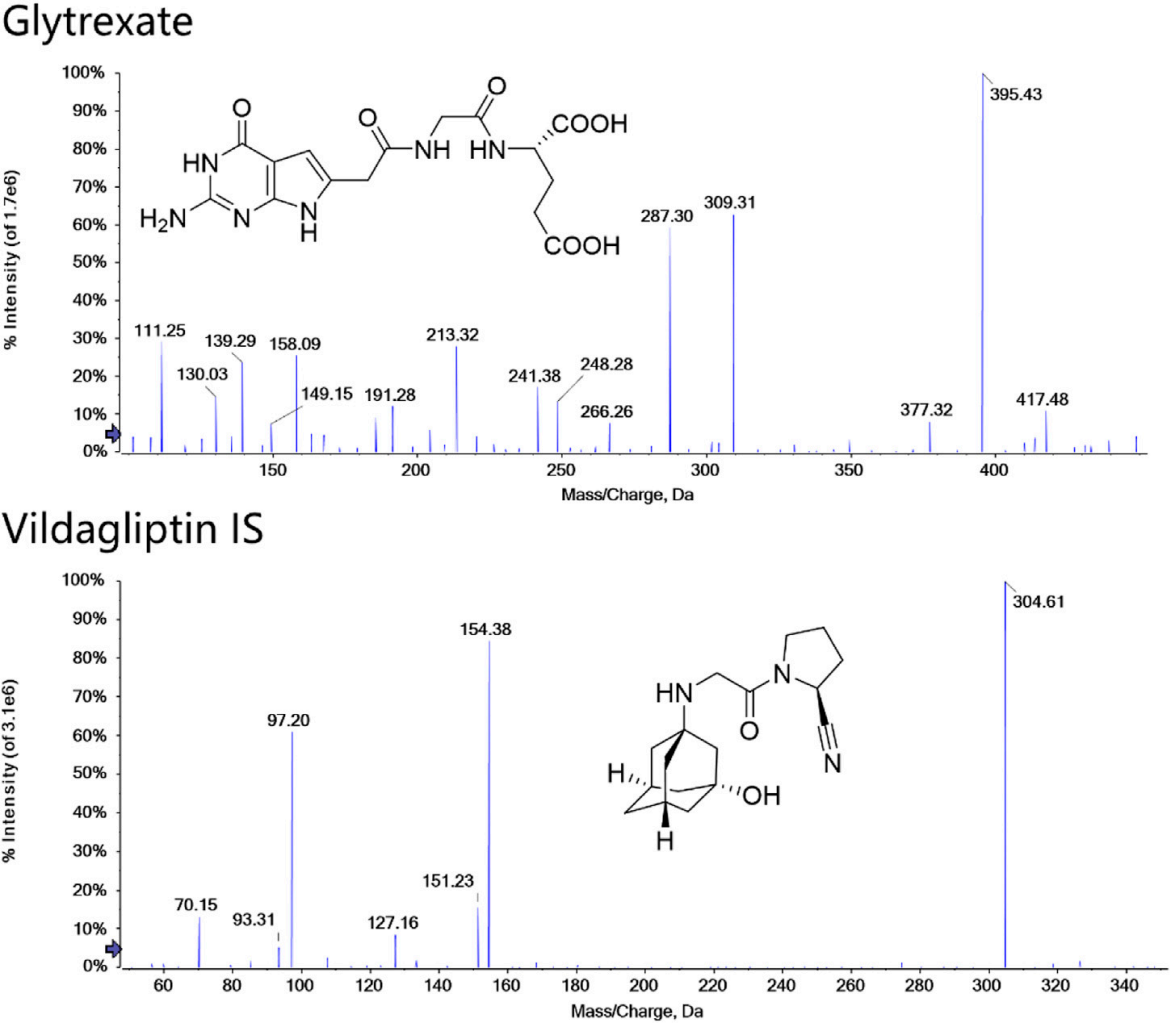
Structures and MS/MS spectra of glytrexate and the IS. MS/MS data were obtained from the respective [M+H]^+^ ions as the precursors for collision-induced dissociation.

Glytrexare (Figure 1, C_15_H_18_N_6_O_7_; >96% purity) was developed by Hebei Medical University (Shijiazhuang, China) (Xing et al., 2017). Vildagliptin (Figure 1, internal standard, IS; 99.5% purity) was obtained from the National Institutes for Food and Drug Control (China). HPLC-grade formic acid was purchased from Dikma Technology (United States). HPLC-grade methanol and acetonitrile were obtained from J.T. Baker (United States). Purified water was obtained from Hangzhou Wahaha Group Co., Ltd. (China). PBS (100 ml, pH 7.4) and human liver microsomes (1 mg/ml) were purchased from Bioreclamationivt Technology Company, United States. Amicon Ultra0.5 ultrafiltration centrifuge tubes were purchased from Millipore, United States (relative molecular weight cutoff: 10,000 Da). Human plasma was purchased from FRESENIUS KABI (Guangzhou, China, H20033707).

Male Sprague‒Dawley rats (250 ± 10 g) for use in the pharmacokinetic study were purchased from the Experimental Animal Center, Hebei Medical University, Shijiazhuang, China and housed under standard temperature, humidity, and light conditions with food (Laboratory Rodent Chow) and water provided *ad libitum*. For the pharmacokinetic studies, animals at the age of 8–10 weeks were weighed. All animals were raised and cared for at 25°C ± 1°C with a relative humidity of 50% ± 10%. Animals were allowed to acclimatize to the laboratory for at least 1 week and were fasted overnight (12 h) with free access to water before experiments. All animal experiments were approved by the Laboratory Animal Ethical and Welfare Committee of Hebei Medical University (IACUC-Hebmu-2022010) and were performed following the Guidelines for the Care and Use of Laboratory Animals of Hebei Medical University.

### 2.2 Instrumentation and analytical conditions

The LC-MS/MS system included an Agilent 1200 high-performance liquid chromatograph (Agilent, United States), a 3200 QTRAP triple quadrupole linear ion trap mass spectrometer (Sciex, United States), and Analyst 1.6.3 data processing software. Glytrexate and IS were detected using an electrospray ionization (ESI) source.

Glytrexate and IS were separated using a Kinetex® 2.6 µm F5 100 Å LC column (100 × 2.1 mm, 2.6 μm). The mobile phase consisted of a mixture of water (A, containing 0.2% formic acid, v/v) and 70% acetonitrile (B, containing 0.2% formic acid, v/v). The gradient elution was 0–1 min, 6–80% B; 1–1.5 min, 80–100% B; 1.5–5 min, 100% B; and 5–6 min, 100–6% B. The column temperature was kept at 35°C with a flow rate of 0.3 ml/min. The injection volume was set to 5 μl.

Detection was performed in multiple reaction monitoring (MRM) mode with positive ion electrospray ionization. Two selective MRM transitions were monitored with *m/z* 395.4→ 191.2 (quantifier) and 395.4→248.2 (qualifier) for glytrexate and 304.6→154.3 (quantifier) and 304.6→97.2 (qualifier) for the IS. The optimized parameters were as follows: the ion spray voltage floating (ISVF), curtain gas pressure, nebulizer gas (GS1) pressure, heater gas (GS2) pressure and ion source temperature (TME) were 5.5 kV, 35 psi, 55 psi, 55 psi, and 550°C, respectively. The adjusted values of declustering potential (DP) and collision energy (CE) were 44 V and 28 eV and 25 eV for glytrexate and 100 V and 25 eV and 40 eV for IS. The structure and product ion mass spectra of glytrexate and the IS are shown in Figure 1.

Quantitation was performed using MultiQuant software with a six-point calibration curve. The MQ4 integration algorithm was used to integrate peaks. The calibration curves were established using linear regression with 1/x^2^ weighting. For the approaches, the peak area ratio of analyte/IS was evaluated for quantitation.

### 2.3 Solution preparation

#### 2.3.1 Preparation of stock solution

Standard stock solutions of glytrexate and IS were dissolved in methanol at the same concentration of 1 mg/ml. The stock solution of vildagliptin was further diluted to obtain a working solution of 100 ng/ml with 20% methanol (1% formic acid, v/v). All the solutions were stored at 4°C until use.

#### 2.3.2 Preparation of standard and quality control samples

Based on a preliminary experiment, we chose to prepare a calibration curve in the range of 20–20000 ng/ml. The standard solution (20, 50,100, 200, 500, 1000, 2000, 5,000, 10,000, 20,000 ng/ml) were prepared by continuously diluting the stock solution with 20% methanol (1% formic acid, v/v). Calibration glytrexate standards of glytrexate were prepared by spiking 5 μl standard working solutions into 50 μl blank rat plasma.

The quality control (QC) samples were prepared by adding glytrexate into the same matrix to reach final concentrations of 2, 5, 200, and 1600 ng/ml (LLOQ, LQC, MQC, and HQC) with an independently prepared standard solution of glytrexate. The standards and QC samples were handled according to the same sample processing steps as unknown samples (Nouman et al., 2015; Toennes et al., 2017; Qiu et al., 2021).

### 2.4 Preparation of plasma samples

Fifty microliters of plasma was spiked with an aliquot of 5 μl IS working solution (100 ng/ml), vortexed with 150 μl of methanol (1% formic acid) for 60 s, and centrifuged at 15,000 r for 10 min at 4°C to precipitate proteins. Then, the supernatant of the samples (165 μl) was accurately transferred into a new 1.5 ml centrifuge tube and evaporated to dryness under nitrogen flow at room temperature. After reconstitution with 50 μl of 20% methanol (1% formic acid), the samples were vortexed and centrifuged at 14,000 rpm for 10 min. Subsequently, 5 μl of the supernatant was injected into the LC-MS/MS system for analysis (Schofield et al., 2016; Chen et al., 2019).

### 2.5 Bioanalytical method validation

The LC-MS/MS method established in this study was validated according to the regulatory guidelines of the ICH guidelines (ICH, 2022). The specific content includes specificity, linearity, precision, accuracy, extraction recovery, matrix effect, stability and dilution integrity (Erdogar et al., 2021).

#### 2.5.1 Specificity

Specificity assessment was performed in which the chromatograms of blank rat plasma samples, samples spiked with glytrexate and IS, and samples collected after oral administration were compared to explore the potential interference of retention times between the analytes. There should be no obvious interference observed at the retention times of the analytes and IS in blank samples and actual biological samples.

#### 2.5.2 Linearity and lower limit of quantification

The linearity of glytrexate was evaluated by plotting the glytrexate/IS peak area ratios versus the glytrexate concentrations. The linearity was obtained from a calibration curve with nine points ranging from 2 to 2000 ng/ml glytrexate in plasma samples and fitted to y = a + bx *via* weighted squares linear regression. The abscissa was the concentration of glytrexate and the ordinate was the ratio of the peak area. The linearity degree is expressed by the correlation coefficient (r).

The lower limit of quantification (LLOQ) was defined as the lowest calibration curve concentration, at which the deviation of accuracy (relative error, RE) was within ±20% and the precision (relative standard deviation, RSD) < 20%. The signal-to-noise ratio (S/N) was not <10.

#### 2.5.3 Accuracy and precision

The accuracy and precision of the established method were measured at four QC levels (LLOQ, LQC, MQC, and HQC) in six replicates on the same day (intraday) and on three consecutive days (interday). The accuracy and precision of the nominal concentration at four QC levels should be within ±15%, and at the LLOQ level should be within ±20%.

#### 2.5.4 Extraction recovery and matrix effect

Extraction recoveries were evaluated by the LLOQ, LQC, MQC, and HQC concentrations of QC samples. The matrix effect was evaluated by comparing the mean peak areas of glytrexate in blank rat plasma samples (*n* = 6) with those in neat solutions at four QC concentrations.

#### 2.5.5 Stability

For short-term and long-term stability, plasma samples (LQC and HQC) were kept at room temperature for 8 h and at −20°C for 14 consecutive days, respectively. To evaluate the freeze-thaw stability, plasma samples (*n* = 6) were investigated through three cycles from −20°C to 25°C, and the stability of the postpreparative samples after 8 h was evaluated in autosampler vials at 4°C. The results were evaluated by calculating the peak area ratio (glytrexate/IS) of the stability samples. If glytrexate is stable in rat plasma, the accuracy (RE) should be within ±15% and the precision (RSD) < 15%.

#### 2.5.6 Dilution integrity

Rat plasma glytrexate concentrations at the high concentration were more than 2000 ng/ml (upper limit of quantification [ULOQ]) at a dose of 2 mg/kg intravenous injection. Plasma samples were prepared at a concentration exceeding the ULQC, and plasma samples were diluted 10-fold, 20-fold, and 50-fold with a blank matrix to fall within the concentration range of the calibration curve.

### 2.6 *In vitro* studies

#### 2.6.1 Stability in plasma, simulated gastrointestinal fluids, and human liver microsomes

Blood was taken from the posterior canthal venous plexus of SD rats and added to a heparin sodium anticoagulant centrifuge tube prepared in advance of the experiment. After 10 min of centrifugation at 3500 rpm, the upper plasma was immediately frozen and stored at −20°C for future use.

Blank artificial gastric fluid: hydrochloric acid (2.75 mol/L, 16.4 ml) was diluted with 800 ml of water, adjusted to the pH 1.3 with 0.1 mol/L hydrochloric acid solution, diluted with water and diluted to 1000 ml.

Artificial gastric fluid: hydrochloric acid (2.75 mol/L, 16.4 ml) was diluted with 800 ml of water, and then 10 g of pepsin was added and shaken well until dissolved. The pH was adjusted to 1.3 with 0.1 mol/L hydrochloric acid solution, and then the artificial gastric fluid solution was diluted with water to 1000 ml.

Blank artificial intestinal fluid: potassium dihydrogen phosphate (6.8 g) was diluted with 500 ml of water, and shaken until dissolved. The pH was adjusted to 6.8 with 0.1 mol/L sodium hydroxide solution, and the solution was diluted with water to 1000 ml.

Artificial intestinal fluid: potassium dihydrogen phosphate (6.8 g) was diluted with 500 ml of water and shaken until dissolved. The pH was adjusted to 6.8 with 0.1 mol/L sodium hydroxide solution. Then, 10 g of trypsin was added, and the appropriate amount of water was added to dissolve the trypsin. After the two solutions were mixed, water was added to dilute the solution to 1000 ml to obtain artificial intestinal fluid.

The test tube containing the drug concentration was incubated in a constant temperature water bath at 37°C. Each sample was obtained at 0, 0.5, 1.0, 1.5, 2.0, and 3.0 h, and the drug concentration was measured.

#### 2.6.2 Study on plasma protein binding rate

The glytrexate stock solution was diluted with 20% methanol (1% formic acid) to three mass concentrations of 50, 100, and 200 μg/ml for use. One hundred microliters of glytrexate solution of the corresponding concentration (3 duplicates each in parallel) was added to 900 μl of rat/human plasma, vortexed and mixed well to prepare plasma samples with plasma concentrations of 5, 10, and 20 μg/ml.

The plasma sample was placed in a constant temperature water bath at 37°C for a certain period to obtain an incubation solution. After the incubation solution was removed, it was placed on ice to maintain a low temperature, transferred to a filtrate tube (500 μl), and placed in a low-temperature refrigerated centrifuge at 4°C and 14,000 rpm for 20 min to obtain filtrate.

The test tube containing the drug concentration was incubated in a constant temperature water bath at 37°C. Each sample was obtained at 10 min, 30 min, 60 min, 120 min, 240 min, and 360 min and the drug concentration before and after ultrafiltration was determined. The plasma protein binding rate (%) = [(C_incubation solution_-C_Ultrafiltrate_)/C_incubation solution_]×100%. GraphPad Prism 8 software was used to perform a one-way analysis of variance on the plasma protein binding rate of glytrexate between different mass concentrations in the same species. The plasma protein binding rate between different species was tested using an independent sample t test, and *p* < 0.05 indicated that the difference was significant.

#### 2.6.3 Sample preparation and processing

Briefly, 500 μl of different simulated gastrointestinal fluids were placed in a 1.5 ml centrifuge tube, and 50 μl of sample solution was added. This mixture was vortexed for 30 s and incubated at 37°C for various times.

The metabolic stability of glytrexate was determined by incubation with human liver microsomes at 37°C. The reaction in the incubation system containing 10 μl of glytrexate (1 mg/ml), 50 μl of liver microsomes (1 mg/ml), and 10 μl of PBS (pH 7.4) was initiated by the addition of 30 μl of NADPH after 5 min of preincubation. The total incubation volume was 100 μl. The reaction was terminated at predefined time points (0, 0.5, 1, 1.5, 2, and 3 h). The incubations were conducted in triplicate.

Fifty microliters of the above solution was accurately drawn at different incubation time points, and 155 μl of ice-cold methanol (1% formic acid) and 5 μl of IS solution were added. The mixture was vortexed for 60 s and centrifuged at 14,000 rpm for 10 min. Then, 165 μl of supernatant was transferred to a new tube and evaporated to dryness under a gentle stream of nitrogen at room temperature. The residue was dissolved in 50 μl 20% methanol (1% formic acid), vortexed for 60 s and centrifuged at 14,000 rpm for 10 min. An appropriate amount of the supernatant was placed into the sample vial, and analyzed. Each experiment was repeated 3 times.

The plasma sample concentration was 1 μg/ml, and the concentration of the other samples was 100 μg/ml.

All samples were analyzed using the established LC‒MS/MS method, and MultiQuant 3.0 software was used for data processing. The glytrexate concentration at each time point was calculated, the remaining percentage was calculated, and the concentration change trend was analyzed.

### 2.7 Pharmacokinetic study

#### 2.7.1 Experimental design of animal studies

Pharmacokinetic and bioavailability studies were carried out in twenty-four male Sprague–Dawley rats. The rats were randomly divided into four groups (*n* = 6). All of them were fasted for 12 h before administration and were allowed to drink freely. Approximately 300 μl of blood was collected from the posterior canthus venous plexus of each rat into heparinized tubes at 0.1, 0.16, 0.25, 0.5, 1, 2, 4, 8, 12, and 24 h after a single oral administration (i.g.) (12.5, 25, and 50 mg/kg) or at 0.03 h, 0.08, 0.25, 0.5, 1, 2, 4, 6, 8, 12, and 24 h after a single i.v. injection (2 mg/kg). The blood samples were processed to obtain the plasma *via* centrifugation at 3500 *rpm* for 10 min and then stored at −80°C until LC–MS/MS analysis.

#### 2.7.2 Statistical analysis

The plasma samples obtained were processed using the method described in Section 2.4 and then analyzed using the established LC–MS/MS method. The data were processed using MultiQuant 3.0 software. According to the results of the blood drug concentration measurement in rats, the noncompartmental model in DAS 3.2.8 software was used to calculate the corresponding pharmacokinetic parameters, including peak time (T_max_), elimination half-life (T_1/2_), area under the drug-time curve (AUC_0-t_, AUC_0-∞_), maximum plasma concentration (C_max_), apparent volume of distribution (V_z_/F), clearance rate (Cl_z_/F), and average residence time (MRT_0-t_). GraphPad Prism 8 software was used to construct the trend chart of the changes in various parameters in plasma samples based on the measurements to analyze the possible pharmacokinetic characteristics of glytrexate.

If glytrexate has linear kinetic characteristics, the absolute bioavailability can be calculated according to the bioavailability formula, Fabs=(AUC_T_·D_iv_)/(AUC_iv_·D_T_) × 100%, where AUC represents the area under the plasma concentration-time curve. The subscripts T and iv represent oral and intravenous injection, respectively, and D represents the administered dose. The data obtained in the study are expressed as the mean values plus or minus the standard deviation.

## 3 Results

### 3.1 Liquid chromatography-tandem mass spectrometry method development and validation

**FIGURE 2:**
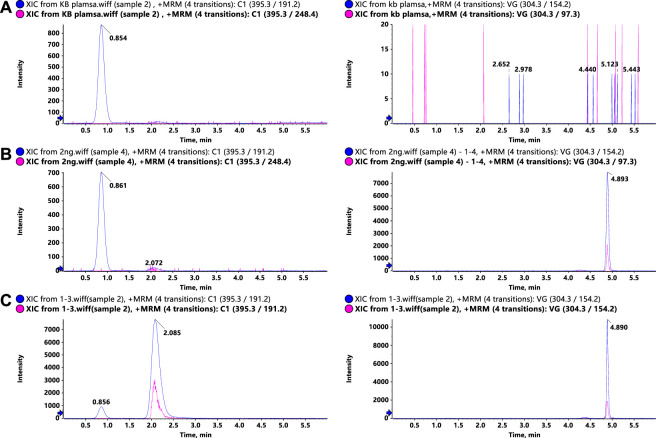
Representative LC–MS/MS chromatograms of glytrexate (left) and the IS (right) in rat plasma. **(A)** Blank rat plasma sample, **(B)** blank rat plasma sample spiked with 2 ng/ml glytrexate, and **(C)** rat plasma sample obtained 2 h after an oral administration of 50 mg/kg glytrexate.

To optimize the MS responses of glytrexate and vildagliptin, both negative and positive ESI modes were used. For the desired sensitivity, the positive mode yielded higher and more stable ion production. Several chromatographic parameters were optimized to achieve suitable chromatographic behavior. We used four columns: ACQUITY UPLC HSS C_18_, Kinetex 2.6 μm XB-C_18_, Synergi 4 μm Fusion-RP, and Kinetex 2.6 μm F5. Due to the high polarity of glytrexate, the preferable retention of glytrexate and vildagliptin was conducted on a Kinetex 2.6 μm F5 column, which could withstand a high proportion of the aqueous phase. To achieve the desired peak shape and ionization, various mobile phase compositions, including water-methanol and water-acetonitrile, were investigated. The final mobile phase contained 0.2% formic acid aqueous and 70% acetonitrile (0.2% formic acid, v/v) and was chosen based on the good peak shape and high sensitivity observed. Figure 2 shows a major interference at 0.8 min in the extracted ion chromatogram (XIC) of the drug candidate. The interference is likely not related to the drug, as it does not have the same area ratio for the peak at 2.1 min (comparing Figures 2B,C). However, this interference could be an endogenous molecule in the plasma. We chose two ion pairs for quantification and detected a difference in retention time between the 0.8 min peak and glytrexate; therefore, we do not think that this interference impairs the quantitative determination.

### 3.2 Sample preparation

Glytrexate and vildagliptin were extracted from plasma samples using both protein precipitation and liquid‒liquid extraction techniques. Glytrexate was difficult to extract from plasma samples using organic solvents due to its high polarity. Thus, plasma samples were analyzed using the protein precipitation method, and methanol was employed as the precipitation agent. The addition of formic acid was investigated and found to produce a higher recovery rate with less interference. The results demonstrated that 1% formic acid greatly enhanced the recovery of glytrexate.

### 3.3 Method validation

The method validation data (individual data points) has been included as Supplementary Material

#### 3.3.1 Specificity

Representative chromatograms of blank rat plasma, blank rat plasma spiked with 2 ng/ml glytrexate, and rat plasma samples at 2 h after an oral administration of 50 mg/kg glytrexate are shown in Figure 2. The retention times of glytrexate and the IS were approximately 2.1 min and 4.8 min, respectively, and the analysis time for each sample was 6 min. The results showed good method selectivity; that is, the endogenous components in the biological samples did not interfere with the retention times of glytrexate or the IS.

#### 3.3.2 Linearity and lower limit of quantification

The linearity of glytrexate in plasma was obtained over a range of 2–2000 ng/ml (y = 0.0008644x+0.00406, r = 0.99992) using a 1/x^2^ weighting based on the peak area ratios of glytrexate to vildagliptin versus glytrexate concentration. The LLOQ was validated to be 2 ng/ml, where the RE and RSD values were within the acceptable limits and S/N > 10.

#### 3.3.3 Accuracy and precision

The precision and accuracy test results are shown in Table 1. The RSDs of intraday and interday precision were 1.65–4.27% and 1.50–11.52%, respectively, and the accuracy RE was −6.64–0.52%. All results indicated that the analytical method was accurate and reliable.

**TABLE 1:** Intraday and interday precision and accuracy for glytrexate in rat plasma (*n* = 6).

Added (ng/ml)	Intraday		Interday	
	Mean±SD (ng/ml)	RSD (%)	RE (%)	RSD (%)
2	1.87 ± 0.04	1.65	−6.64	3.98
5	4.74 ± 0.11	1.83	−5.31	4.89
200	193.91 ± 9.46	3.04	−3.05	11.52
1600	1608.31 ± 65.10	4.27	0.52	1.50

All glytrexate values are listed as the mean ± SD.

#### 3.3.4 Extraction recovery and matrix effect

According to the QC sample preparation method, 4 types of concentration quality control samples, each with 6 samples were prepared, and instrumental analysis and determination were performed to obtain the peak area (C). Water was used to replace plasma, and 4 quality control samples were prepared with the QC sample preparation method, 6 samples of each, for instrumental analysis and determination, and the peak area (A) was obtained. After the blank rat plasma was processed following the QC sample preparation method, 2, 5, 200, and 1600 ng/ml glytrexate and 100 ng/ml vildagliptin solution were added to prepare samples (*n* = 6) of corresponding concentrations, and the peak area (B) was obtained *via* instrumental analysis. The extraction recovery rate formula was C/B × 100%, and the matrix effect formula was B/A × 100%. The test results presented in Tables 2, 3 show that the extraction recovery rates for glytrexate and vildagliptin in rat plasma were 96.32–103.10% (RSD 10.53–13.62%) and 101.20% (RSD 11.14%), respectively. The matrix effects of glytrexate and the internal standard vildagliptin in rat plasma were 103.32–108.28% (RSD 3.31–5.27%) and 104.37% (RSD 7.01%), respectively. These results revealed that the recoveries of glytrexate in rat plasma were within an acceptable range, and no notable endogenous interferences were observed in the detection of glytrexate in the rat biosamples.

**TABLE 2:** Extraction recoveries and matrix effects of glytrexate and the IS in rat plasma (*n* = 6).

Compounds	Spiked conc. (ng/ml)	Extraction recoveries (%)	RSD (%)	Matrix effects (%)	RSD (%)
Glytrexate	2	103.10 ± 11.10	10.77	108.28 ± 4.60	4.25
	5	102.71 ± 10.89	10.61	105.72 ± 5.48	5.19
	200	96.32 ± 13.12	13.62	103.32 ± 3.42	3.31
	1600	97.02 ± 10.22	10.53	106.39 ± 5.60	5.27
IS	100	101.20 ± 11.27	11.14	104.37 ± 7.31	7.01

All glytraxate values are listed as the mean ± SD.

**TABLE 3:** Stability of glytrexate in rat plasma (*n* = 6).

	Spiked conc. (ng/ml)	
	5	1600
At room temperature for 8 h in rat plasma		
Mean (*n* = 6)	4.49 ± 0.40	1586.82 ± 103.89
% Deviation	−10.23	−0.82
% CV	8.98	6.55
At 4°C for 8 h in rat plasma		
Mean (*n* = 6)	4.43 ± 0.21	1566.21 ± 100.13
% Deviation	−11.50	−2.11
% CV	4.77	6.39
After three freeze‒thaw cycles in rat plasma		
Mean (*n* = 6)	4.89 ± 0.68	1571.07 ± 105.54
% Deviation	−2.27	−1.81
% CV	13.85	6.72
At −20°C for 14 days in rat plasma		
Mean (*n* = 6)	4.68 ± 0.40	1567.82 ± 112.14
% Deviation	−6.40	−2.01
% CV	8.49	7.15

All glytrexate values are listed as the mean ± SD.

#### 3.3.5 Stability

The stabilities of glytrexate in rat plasma were assessed after storage at room temperature for 8 h and at 4°C for 8 h, after freeze‒thaw cycles, and after 14 days of long-term storage. The data are shown in Table 3. During the storage process, the samples were protected from light and sealed, and no obvious degradation was detected during the sample preparation procedures and storage conditions, indicating that the storage conditions and preparation method were appropriate for routine analysis.

#### 3.3.6 Dilution integrity

Through the analysis of 10,000 ng/ml glytrexate plasma samples diluted 10 times, 20 times, and 50 times, the dilution reliability was evaluated according to the precision and accuracy results. As shown in Table 4, the precision and accuracy of the diluted sample were within ± 15%, which meets the requirements for biological sample analysis.

**TABLE 4:** Dilution integrity of glytrexate in rat plasma (*n* = 6).

Preparation concentration (ng/ml)	Dilution multiple	Measured concentration (ng/mL)	RSD (%)	Accuracy (%)
1000	10	1088.31 ± 63.45	5.83	108.83
500	20	511.68 ± 10.51	2.05	102.34
200	50	200.63 ± 13.21	6.59	100.32

All glytrexate values are listed as the mean ± SD.

### 3.4 *In vitro* studies

#### 3.4.1 Stability in plasma, simulated gastrointestinal fluid and human liver microsomes

**FIGURE 3:**
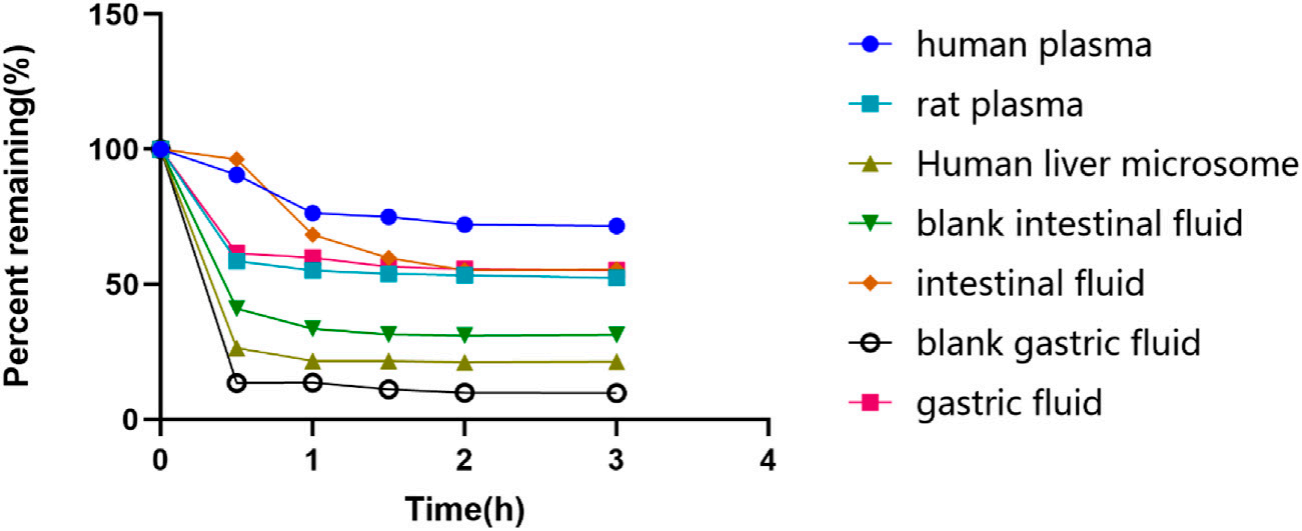
The remaining glytrexate percentages over time. Stability of glytrexate following incubation in human liver microsomes, rat plasma, human plasma, blank intestinal fluid, intestinal fluid, blank gastric fluid, and gastric fluid at 37°C for 0, 0.5, 1, 1.5, 2, and 3 h. The glytrexate changes after incubation with different fluids. The percentage of glytrexate was calculated based on the amount remaining. All glytrexate values are listed as the mean values.

The glytrexate concentrations after 0, 0.5, 1.0, 1.5, 2.0, and 3.0 h of incubation at 37°C in different substrates are shown in Figure 3. Glytrexate degraded slowly after the first 0.5 h in all test fluids, with a significant amount remaining in the intestinal fluid. After 2 h of incubation, the remaining percentage of glytrexate in each sample remained unchanged. The graph shows that after 3 h of incubation, the greatest amount of remaining glytrexate was found in human plasma.

#### 3.4.2 Plasma protein binding rate

**FIGURE 4:**
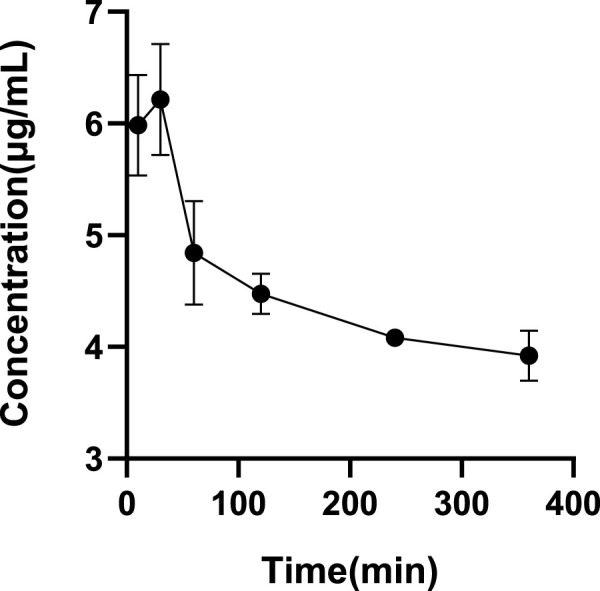
Determination of the optimal incubation time. All glytrexate values are listed as the mean value ± SD. Bars represent SD.

The ultrafiltrate concentrations after incubation for 10 min, 30 min, 60 min, 120 min, 240 min, and 360 min were tested. Figure 4 shows that the drug concentration was at its highest at 30 min among all the incubation times. As a result, we decide that 30 min is the ideal incubation period. Table 5 shows the plasma protein binding rates of glytrexate in rat and human plasma were 5.29–18.68% and 19.23–24.61%, respectively.

**TABLE 5:** Plasma protein binding rate of glytrexate in rat and human plasma (mean ± SD, *n* = 3).

Concentration (μg/ml)	Rat plasma (%)	Human plasma (%)
5	13.68 ± 4.44	24.22 ± 2.42
10	18.68 ± 2.53	24.61 ± 2.57
20	5.29 ± 4.67	19.33 ± 5.11

All glytrexate values are listed as the mean ± SD.

### 3.5 *In vivo* pharmacokinetic study

**FIGURE 5:**
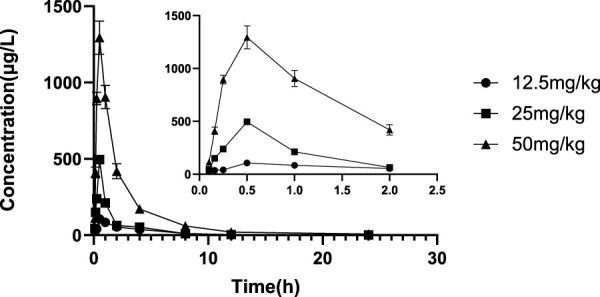
Mean plasma concentration-time profile of glytrexate after 12.5, 25, and 50 mg/kg glytrexate was administered to rats (*n* = 6). All the glytexate PK values are listed as the mean ± SD. Each mean ± SD (*n* = 6) is represented by each point and vertical bar, respectively. Bars represent SD.

**FIGURE 6:**
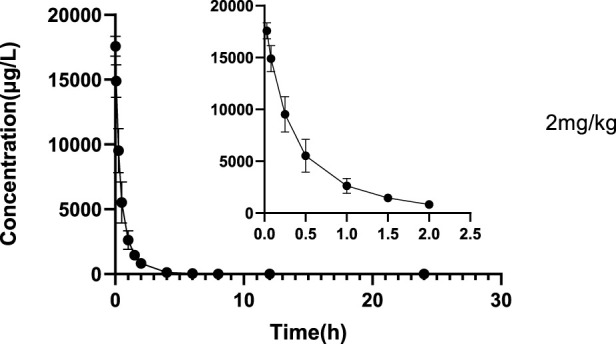
Mean plasma concentration-time profile of glytrexate after 2 mg/kg glytrexate was injected into the tail vein of rats (*n* = 6).

**FIGURE 7:**
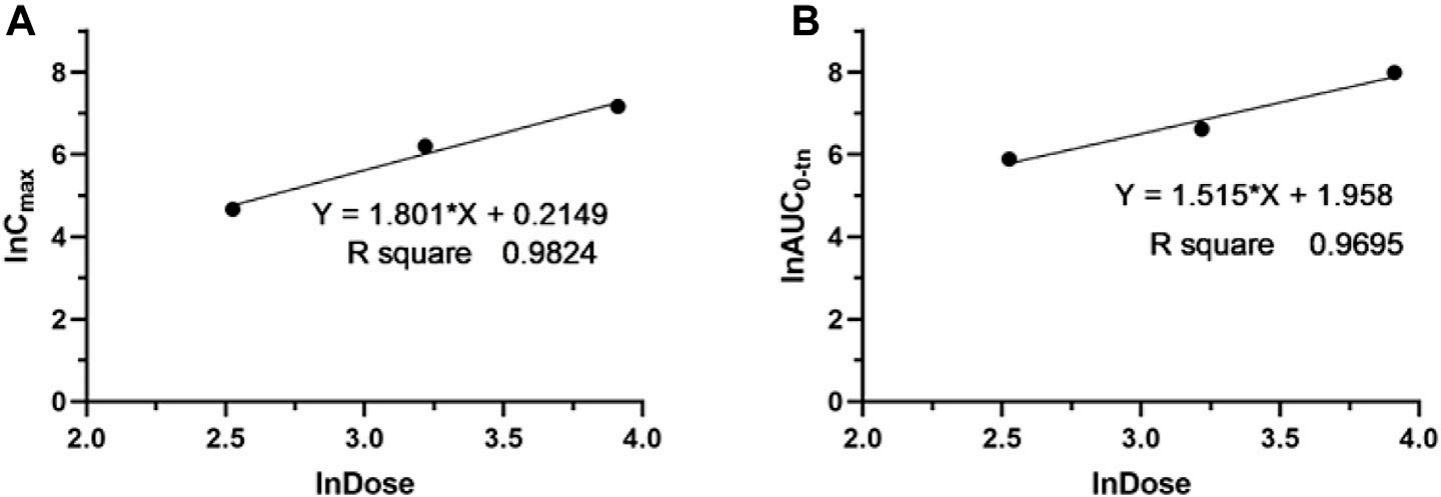
Dose proportionality of C_max_ **(A)** and AUC_0-t_ **(B)** for glytrexate in plasma after a single oral administration of glytrexate at doses of 12.5, 25, and 50 mg/kg.

The validated LC‒MS/MS method was further applied to determine the pharmacokinetic behavior of glytrexate in rats. The mean plasma concentration-time curves following i. g. administration of 12.5, 25, or 50 mg/kg and a single i.v. administration of 2 mg/kg are presented in Figures 5, 6, respectively. Figure 5 shows the peak blood concentration of glytrexate at 0.5 h after a single oral administration (i.g.) (12.5, 25, and 50 mg/kg). Based on the absence of absorption during intravenous administration, Figure 6 shows the highest plasma concentration at 0.03 h after a single i.v. injection (2 mg/kg). The pharmacokinetic parameters of glytrexate are summarized in Table 6. Glytrexate reached the maximum plasma concentration (C_max_) at approximately 0.5 h. The T_1/2_ after oral administration of 12.5, 25, or 50 mg/kg was 2.15 ± 0.16, 2.25 ± 0.093, and 2.66 ± 0.15, respectively. The AUC_0-t_ of oral administration of 12.5, 25, or 50 mg/kg was 362.32 ± 24.55, 749.87 ± 30.24, and 2959.53 ± 189.42, respectively. Figure 7 shows that the AUC and C_max_ both increased with increasing oral administration dose, the average residence time did not increase with increasing dose (p > 0.05), and the T_max_ was both 0.5 h, which means that glytrexate meets linear pharmacokinetic characteristics. The half-life in the high-dose group was higher than that in the low-medium-dose group, and the elimination rate and apparent volume of distribution were lower in the high-dose group than in the low-medium-dose group. The oral bioavailability of glytrexate in rats was 0.57–1.15% according to the calculation results.

**TABLE 6:** Pharmacokinetic parameters of glytrexate following single oral and intravenous administrations (*n* = 6).

Parameter	Oral			Intravenous
	12.5 mg/kg	25 mg/kg	50 mg/kg	2 mg/kg
C_max_ (μg/L)	106.53 ± 12.12	496.05 ± 19.77	1294.38 ± 108.84	17575.89 ± 766.73
T_max_ (h)	0.5	0.5	0.5	—
T_1/2_ (h)	2.15 ± 0.16	2.25 ± 0.093	2.66 ± 0.15	2.78 ± 0.26
AUC_0-tn_ (μg h/L)	362.32 ± 24.55	749.87 ± 30.24	2959.53 ± 189.42	10223.68 ± 1712.53
AUC_0-∞_ (μg h/L)	370.46 ± 25.98	750.19 ± 30.20	2963.18 ± 189.91	10222.21 ± 1712.57
V/F (L/kg)	104.72 ± 6.71	108.42 ± 7.70	64.83 ± 4.53	0.80 ± 0.15
Cl/F (L/h/kg)	33.89 ± 2.57	33.37 ± 1.32	16.94 ± 1.18	0.20 ± 0.03
MRT_0-t_ (h)	3.12 ± 0.14	2.95 ± 0.16	3.16 ± 0.096	0.91 ± 0.05
F (%)	0.57 ± 0.08	0.59 ± 0.06	1.15 ± 0.07	—

T_1/2_, elimination half-life; T_max_, time of maximum concentration; C_max_, maximum concentration; AUC_0-tn_, area under the plasma concentration–time curve (AUC) computed from time zero to the time of the last positive Y value; AUC_0-∞_, AUC, from time zero extrapolated to infinity; MRT_0-t_, mean residence time when the drug concentration is based on values up to and including the last measured concentration; V/F, the volume of distribution during the terminal phase of pseudoequilibrium scaled by bioavailability; Cl/F, body clearance scaled by bioavailability. All the glytrexate PK, values are listed as the mean value ± SD. F, bioavailability. All the glytrexate PK, values are listed as the mean value ± SD., Each mean ± SD (*n* = 6) is represented by each point and vertical bar, respectively.

## 4 Discussion

In our previous study, possible anticancer drugs targeting both thymidylate and purine nucleotide biosynthesis were investigated, and glytrexate was one of a series of compounds identified as a multitarget inhibitors of TS, GARFTase, and AICARFTase and found to have antiproliferative effects in a series of tumor cell lines including KB, SW620, and MCF7. Its inhibitory action on KB tumor cells was at a nanomolar level (IC_50_ 0.078 nM), and its efficacy was 140-fold higher than the positive control drug pemetrexed (IC_50_ 0.07 μM) (Xing et al., 2017). According to reports, the cellular uptake of 6-substituted pyrrolo [2,3-d] pyrimidines, depend on multiple (folate) transporters like folate receptor alpha (FRα), FRβ, reduced folate carrier (RFC), and proton coupled folate transporter (PCFT). And they found that the role of folylpolyglutamate synthetase (FPGS) in the retention of 6-substituted pyrrolo [2,3-d] pyrimidines intracellularly (Dekhne et al., 2020; Golani et al., 2020; Wallace-Povirk et al., 2021; Wallace-Povirk et al., 2022). Thus, this hypersensitivity maybe explained by the fact that KB cells express high amount of FRα, which may serve as cellular internalization route for glytrexate.

In the preclinical phase, pharmacokinetic studies are a prerequisite to guarantee that the tested drugs have appropriate drug-like properties. Pharmacokinetic profiles and parameters obtained in rats are helpful for the subsequent preclinical and clinical development of glytrexate. Pharmacokinetic properties are crucial for drug discovery, preclinical development and lead identification, as well as for dosage regimen design and drug formulation development.

Based on the stability results, glytrexate is more stable in human plasma than in human liver microsomes and artificial gastrointestinal fluid. Glytrexate did not remain stable at the pH of the artificial gastrointestinal fluid. Glytrexate degraded quickly during the first 0.5 h in human liver microsomes, and it is speculated that it has a relatively strong first-pass action and subjected to a rapid metabolic reaction. After 0.5 h of incubation with human liver microsomes, the remaining percentage of glytrexate showed no significant change, indicating that a balanced state had been reached. Glytrexate was evaluated for its metabolic stability in human liver microsomes and showed good microsomal stability. By linearly regressing the natural logarithm of the amount of medication remaining at each time point against the incubation period, the kinetic parameter half-life (t_1/2_) was obtained (T_1/2_ = 109.85 min > 60 min). Good *in vitro* human hepatic microsomal metabolic stability was demonstrated, and thus, glytrexate can be further evaluated for *in vivo* pharmacokinetics.

The drug‒drug interactions (DDIs), pharmacodynamics, and pharmacokinetic properties of a drug are closely related to its reversible binding to plasma or serum proteins. Variations in plasma protein content may affect the pharmacokinetics exposure of unbound drugs, leading to alterations in clinical outcomes (Huang et al., 2022). The plasma protein binding rate for glytrexate in human plasma was higher than that in rat plasma, and the plasma protein binding rate in both rats and humans was less than 30%, as shown in Table 5, indicating that the compound binds less to plasma proteins. Pemetrexed has a human plasma protein binding rate of 81% (Robinson et al., 2004). It is usually used in combination with cisplatin to enhance the response rate (Paz-Ares et al., 2003; Purcell and Ettinger, 2003). The human plasma binding rate of glytrexate is nearly 25%, indicating that glytrexate is more likely to exist in a free state, and can more easily permeate the membrane and exert its effect.

The results showed that the AUC and C_max_ both increased with increasing dose, the average residence time did not increase with increasing dose (*p* > 0.05), and the T_max_ was 0.5 h, which means that glytrexate meets linear pharmacokinetic characteristics (Ma et al., 2022). The half-life in the high-dose group was higher than that of the low-medium-dose group, and the elimination rate and apparent volume of distribution in the high-dose group were lower than those in the low-medium-dose group. After oral administration, the T_max_ (0.5 h) and the Vz/F (104.724 ± 6.709, 108.42 ± 7.698, 64.832 ± 4.529 L/kg) values suggested that glytrexate is rapidly absorbed and widely distributed in tissues (Elbadawy et al., 2019). Compared with rat liver blood flow (4.80 L/h/kg), glytrexate exhibited higher CLz/F values (33.891 ± 2.565, 33.369 ± 1.321, 6.937 ± 1.178 L/h/kg), indicating extrahepatic elimination of glytrexate (Huang et al., 2021). Because glytrexate is considerately soluble in water, it may easily excreted by the kidney. While the kidneys express high levels of FRα (Parker et al., 2005), which may bind/resorb glytrexate, maybe cause the potential kidney secretion of glytrexate. In comparison to the pharmacokinetic parameters of pemetrexed in rats, the area under the plasma concentration-time curve (AUC) after oral administration of pemetrexed (20 mg/kg, AUC = 7.55 ± 0.938 μg⋅h/mL) was approximately 12-fold greater than observed for oral glytrexate (25 mg/kg, AUC = 749.87 ± 30.24 μg⋅h/L). The oral bioavailability of pemetrexed in rat was 12.0 ± 1.45% (Pangeni et al., 2018), while that of glytrexate was 0.57–1.15% according to the calculation results. Thus, glytrexate appears to have lower bioavailability than pemetrexed. The intestinal transporter PCFT plays a major role in the bioavailability of the antifolate (Hou et al., 2022). The limited bioavailability of glytrexate maybe caused by it is a poor substrate for PCFT. However, the area under the plasma concentration-time curve (AUC) after intravenous administration of glytrexate (2 mg/kg, AUC = 10,223.68 ± 1712.53 μg⋅h/L) was approximately 1.63-fold greater than that after pemetrexed administration (10 mg/kg, AUC = 31.3 ± 5.34 μg⋅h/mL). The T_1/2_ after intravenous administration of glytrexate (*T*_1/2_ = 2.78 ± 0.26 h) was approximately 5-fold greater than that after the pemetrexed administration (*T*_1/2_ = 0.574 ± 0.071 h) (Pangeni et al., 2018). The investigation revealed that the intravenous half-life, AUC, and C_max_ of glytrexate were higher than those of pemetrexed. Pemetrexed is generally produced as an injection preparation. This provides ideas for the development of glytrexate formulations. Therefore, glytrexate injection has clinical application prospects compared to oral administration. Importantly, the pharmacokinetic parameters provide valuable references for the further study of glytrexate, especially for the analysis of liver microsomal metabolites, the development of intravenous dosage forms, tissue distribution, and excretion.

## 5 Conclusion

In summary, for the first time, a rapid and sensitive LC‒MS/MS bioanalysis method for quantification of glytrexate was developed and fully validated. The analytical method was applied to investigate the stability, plasma protein binding rate, and pharmacokinetics of glytrexate in rats after oral and intravenous administration. Compared with liver microsomes and artificial gastrointestinal fluid, glytrexate is more stable in plasma. Oral administration results were consistent with linear pharmacokinetics. Moreover, these glytrexate data can be employed to design clinical dosage regimens, enhance knowledge of underlying mechanisms, and promote novel drug development.

## Data Availability

The original contributions presented in the study are included in the article/Supplementary Material; further inquiries can be directed to the corresponding authors.
