# Supplementary material for: Pharmacokinetics, bioavailability, and plasma protein binding study of glytrexate, a novel multitarget antifolate
Source: Front Pharmacol. 2022 Oct 4;13:1001308. doi: 10.3389/fphar.2022.1001308 (PMC9577195; doi:10.3389/fphar.2022.1001308)
Supplement: Supplementary file 1 [file DataSheet1.docx]

Pharmacokinetics, bioavailability, and plasma protein binding study of glytrexate, a novel multitarget antifolate

Supplementary Material

**Supplementary Tables: 4**

Table S1 Intraday and interday precision and accuracy for glytrexate in rat plasma (n=6)

| Run | Added Concentration(ng/mL) | | | |
| --- | --- | --- | --- | --- |
|  | 2.00 | 5.00 | 200.0 | 1600.0 |
| 1 | 1.82 | 4.63 | 186.72 | 1535.53 |
|  | 1.93 | 4.76 | 201.68 | 1547.58 |
|  | 1.85 | 4.71 | 213.47 | 1587.85 |
|  | 1.85 | 4.70 | 211.39 | 1748.40 |
|  | 1.88 | 4.73 | 209.68 | 1536.78 |
|  | 1.88 | 4.75 | 203.64 | 1626.09 |
|  | | | | |
| 2 | 1.84 | 4.87 | 187.33 | 1650.90 |
|  | 1.89 | 4.91 | 193.50 | 1704.59 |
|  | 1.93 | 4.85 | 187.68 | 1535.53 |
|  | 1.93 | 4.78 | 188.96 | 1547.58 |
|  | 1.90 | 4.86 | 186.31 | 1587.85 |
|  | 1.90 | 4.83 | 186.78 | 1649.06 |
|  | | | | |
| 3 | 1.83 | 4.61 | 188.29 | 1568.08 |
|  | 1.81 | 4.44 | 186.99 | 1627.10 |
|  | 1.86 | 4.80 | 189.39 | 1582.51 |
|  | 1.83 | 4.64 | 190.33 | 1600.56 |
|  | 1.86 | 4.75 | 189.79 | 1720.16 |
|  | 1.84 | 4.78 | 188.35 | 1593.48 |
| n | 18 | 18 | 18 | 18 |
| Mean(ng/mL) | 1.87 | 4.74 | 193.91 | 1608.31 |
| SD | 0.04 | 0.11 | 9.46 | 65.10 |
| RE(%) | -6.64 | -5.13 | -3.05 | 0.52 |
| Intraday RSD(%) | 1.65 | 1.83 | 3.04 | 4.27 |
| Interday RSD(%) | 3.98 | 4.89 | 11.52 | 1.50 |

Table S2 Extraction recoveries and matrix effects of glytrexate and IS in rat plasma (n=6)

| Added Concentration (ng/mL) | Peak area | | | Extraction recoveries (%) | Matrix effect (%) |
| --- | --- | --- | --- | --- | --- |
|  | C | B | A |  |  |
| 2 | 26.66 | 29.08 | 26.90 | 89.59 | 105.79 |
|  | 29.99 | 28.81 | 27.03 | 100.77 | 104.83 |
|  | 35.35 | 30.19 | 28.82 | 118.78 | 109.84 |
|  | 31.43 | 28.95 | 26.23 | 105.60 | 105.34 |
|  | 27.56 | 32.14 | 27.90 | 92.60 | 116.93 |
|  | 33.11 | 29.40 | 28.03 | 111.24 | 106.96 |
| Mean | 30.68 | 29.76 | 27.49 | 103.10 | 108.28 |
| SD | 3.30 | 1.26 | 0.94 | 11.10 | 4.60 |
| RSD(%) | 10.77 | 4.25 | 3.40 | 10.77 | 4.25 |
| 5 | 77.84 | 72.61 | 61.26 | 109.82 | 108.31 |
|  | 81.58 | 70.36 | 73.66 | 115.10 | 104.95 |
|  | 64.31 | 65.61 | 64.62 | 90.74 | 97.88 |
|  | 70.92 | 76.78 | 68.97 | 100.06 | 114.53 |
|  | 63.51 | 70.04 | 63.43 | 89.61 | 104.47 |
|  | 78.62 | 69.87 | 70.30 | 110.92 | 104.22 |
| Mean | 72.80 | 70.88 | 67.04 | 102.71 | 105.72 |
| SD | 7.72 | 3.68 | 4.70 | 10.89 | 5.48 |
| RSD(%) | 10.61 | 5.19 | 7.01 | 10.61 | 5.19 |
| 200 | 806.91 | 1015.42 | 1003.57 | 82.89 | 100.18 |
|  | 813.47 | 960.28 | 855.63 | 83.56 | 101.93 |
|  | 863.78 | 943.59 | 939.20 | 88.73 | 100.15 |
|  | 985.99 | 966.13 | 976.69 | 101.29 | 102.55 |
|  | 1046.50 | 943.84 | 1015.74 | 107.50 | 107.78 |
|  | 1109.18 | 1011.50 | 862.01 | 113.94 | 107.36 |
| Mean | 937.64 | 973.46 | 942.14 | 96.32 | 103.32 |
| SD | 127.73 | 32.26 | 69.71 | 13.12 | 3.42 |
| RSD(%) | 13.62 | 3.31 | 7.40 | 13.62 | 3.31 |
| 1600 | 9761.36 | 12404.78 | 10470.41 | 82.77 | 111.90 |
|  | 12363.97 | 12585.78 | 10400.25 | 104.83 | 113.53 |
|  | 11967.01 | 11584.89 | 11204.04 | 101.47 | 104.50 |
|  | 10124.65 | 11753.97 | 12921.84 | 85.85 | 106.03 |
|  | 12647.53 | 11547.10 | 10259.86 | 107.24 | 104.16 |
|  | 11789.14 | 10886.62 | 11259.10 | 99.96 | 98.20 |
| Mean | 11442.28 | 11793.86 | 11085.92 | 97.02 | 106.39 |
| SD | 1204.94 | 621.22 | 994.58 | 10.22 | 5.60 |
| RSD(%) | 10.53 | 5.27 | 8.97 | 10.53 | 5.27 |
| IS | 7932.24 | 6769.62 | 7313.20 | 112.27 | 100.00 |
|  | 7879.21 | 7239.37 | 7975.94 | 111.52 | 106.94 |
|  | 7733.65 | 7857.75 | 6420.44 | 109.46 | 116.08 |
|  | 6615.15 | 6672.11 | 5932.04 | 93.63 | 98.56 |
|  | 6067.85 | 6547.76 | 6845.99 | 85.89 | 96.73 |
|  | 6670.32 | 7303.53 | 6128.79 | 94.41 | 107.89 |
| Mean | 7149.74 | 7065.02 | 6769.40 | 101.20 | 104.37 |
| SD | 796.43 | 494.95 | 773.39 | 11.27 | 7.31 |
| RSD(%) | 11.14 | 7.01 | 11.42 | 11.14 | 7.01 |

According to the QC sample preparation method, 4 types of concentration quality control samples, each with 6 samples were prepared, and instrumental analysis and determination were performed to obtain the peak area (C). Water was used to replace plasma, and 4 quality control samples were prepared with the QC sample preparation method, 6 samples of each, for instrumental analysis and determination, and the peak area (A) was obtained. After the blank rat plasma was processed following the QC sample preparation method, 2, 5, 200, and 1600 ng/mL glytrexate and 100 ng/mL vildagliptin solution were added to prepare samples (n=6) of corresponding concentrations, and the peak area (B) was obtained via instrumental analysis. The extraction recovery rate formula was C/B × 100%, and the matrix effect formula was B/A × 100%.

Table S3 Stability of glytrexate in rat plasma (n=6)

| Added Concentration (ng/mL) | Autosampler stability | Bench stability | Three freeze–thaw cycles | Long-term stability |
| --- | --- | --- | --- | --- |
| 5 | 4.08 | 4.28 | 4.11 | 4.38 |
|  | 4.21 | 4.19 | 4.64 | 4.26 |
|  | 5.01 | 4.38 | 5.27 | 4.52 |
|  | 4.33 | 4.36 | 4.16 | 5.11 |
|  | 4.98 | 4.77 | 5.66 | 4.58 |
|  | 4.32 | 4.57 | 5.48 | 5.23 |
| n | 6 | 6 | 6 | 6 |
| Mean(ng/mL) | 4.49 | 4.43 | 4.89 | 4.68 |
| SD | 0.40 | 0.21 | 0.68 | 0.40 |
| CV(%) | 8.98 | 4.77 | 13.85 | 8.49 |
| RE(%) | -10.23 | -11.50 | -2.27 | -6.40 |
| 1600 | 1671.27 | 1672.13 | 1576.64 | 1571.33 |
|  | 1485.49 | 1416.67 | 1718.95 | 1766.25 |
|  | 1733.12 | 1653.65 | 1645.62 | 1543.41 |
|  | 1621.13 | 1597.31 | 1508.23 | 1537.23 |
|  | 1487.89 | 1478.13 | 1415.53 | 1419.65 |
|  | 1522.03 | 1579.36 | 1561.42 | 1569.03 |
| n | 6 | 6 | 6 | 6 |
| Mean(ng/mL) | 1586.82 | 1566.21 | 1571.07 | 1567.82 |
| SD | 103.89 | 100.13 | 105.54 | 112.14 |
| CV(%) | 6.55 | 6.39 | 6.72 | 7.15 |
| RE(%) | -0.82 | -2.11 | -1.81 | -2.01 |

Table S4 Dilution integrity of glytrexate in rat plasma (n=6)

|  | Concentration (ng/mL) | | |
| --- | --- | --- | --- |
|  | 1000 | 500 | 200 |
|  | 1071.45 | 494.34 | 202.33 |
|  | 1064.27 | 523.31 | 209.06 |
|  | 1148.29 | 504.31 | 187.41 |
|  | 1161.21 | 515.76 | 182.58 |
|  | 1097.63 | 517.68 | 205.09 |
|  | 986.99 | 514.67 | 217.32 |
| Mean | 1088.31 | 511.68 | 200.63 |
| SD | 63.45 | 10.51 | 13.21 |
| RSD(%) | 5.83 | 2.05 | 6.59 |
| Accuracy(%) | 108.83 | 102.34 | 100.32 |
